# Supplementary material for: Acceptability of a Patient Portal (Opal) in HIV Clinical Care: A Feasibility Study
Source: J Pers Med. 2021 Feb 16;11(2):134. doi: 10.3390/jpm11020134 (PMC7920437; doi:10.3390/jpm11020134)
Supplement: Supplementary file 1 [file jpm-11-00134-s001.zip › S1_PLWH_Survey.pdf]

OPAL Adaptation for Patients

Please complete the survey below.

Thank you so much!

Date	________________________
What is your date of birth?	________________________
Age	________________________
What is your gender (select all that apply)?	☐ Female<br>☐ Male<br>☐ Trans<br>☐ Other
If other, please specify:	________________________
With respect to your sexual orientation, how do you currently identify? (Check all those that apply)	☐ Heterosexual / straight<br>☐ Lesbian<br>☐ Gay<br>☐ Bisexual<br>☐ Other (Queer, two-spirited, etc.)<br>☐ Unsure
If other, please specify:	________________________
What is your marital status?	☐ Single<br>☐ Married or Common-law<br>☐ Divorced<br>☐ Widow(er)
If a refugee or immigrant - Please add your: Year of entry in Canada, and Country of Origin:	________________________
What ethnic group(s) or family background(s) do you identify with? (Check all that apply)	☐ Caucasian or White<br>☐ Black, African, or Caribbean<br>☐ Asian or Pacific Islander<br>☐ Indian or South Asian<br>☐ North African or Middle Eastern<br>☐ Aboriginal, First Nations, or Métis<br>☐ Latino, Latin American, Hispanic<br>☐ Other
If other, please specify:	________________________

What is the highest level of education that you have completed?	☐ No formal Education<br>☐ Elementary<br>☐ Some high school, but did not graduate<br>☐ High school diploma or a high school equivalency certificate<br>☐ Trade, vocational, or technical diploma certificate<br>☐ Some post-secondary education but no certificates or diploma<br>☐ University, college diploma, or certificate less than a Bachelor's degree<br>☐ Bachelor's degree<br>☐ Graduate (PhD or Masters) or professional degree (medical, law, etc.)<br>☐ Other
If other, please specify	________________________________
What is your current employment status? (check all that apply)	☐ Student<br>☐ Part-time employment<br>☐ Full-time employment<br>☐ Unemployed<br>☐ Homemaker<br>☐ Retired<br>☐ Sick leave or work-related illness / injury leave<br>☐ Long term disability
What was your total income for last year, from all paid work and other sources before taxes and other deductions?	☐ $0 or No income<br>☐ $1-$19 999<br>☐ $20 000 - $39 999<br>☐ $40 000 - $59 999<br>☐ $60 000 - $79 999<br>☐ $80 000 - $99 999<br>☐ $100 000 or more
What is the first language that you learned?	________________________________
What is your preferred language of communication at the service where you receive HIV care?	☐ French<br>☐ English<br>☐ Other
If other, please specify:	________________________________
How much time in minutes, does it take you to travel from your home to the service where you receive HIV care?	________________________________
What mode of transportation do you use to go to the service where you receive HIV care?	☐ On foot / walking<br>☐ Car<br>☐ Public transit (bus, metro, train, etc.)<br>☐ Bicycle<br>☐ Other

If other, please specify:

____

When did you learn you were HIV-positive? (MM/YYYY)

____

Do you currently use any of the following smart devices? (Check all that apply)

- ☐ Smartphone
- ☐ iPod, Phablet
- ☐ Tablet
- ☐ Smartwatch
- ☐ Computer, Laptop
- ☐ Other
- ☐ I do not use any of these devices

If other, please specify

____

Do you currently use an application (app) on a smart device in relation to your health or HIV care?

- ◯ Yes
- ◯ No

If yes, please specify:

____

How much experience do you have with health-related applications(apps) on smart devices?

- ☐ None
- ☐ Very Little
- ☐ Average
- ☐ Quite Extensive
- ☐ Very Extensive

A patient portal is a secure website or application (app) that gives you access to your medical records, as well as other services (for example, a mobile check-in function and messaging with care providers).

Your medical records include: your diagnosis; personal and medical information; a list of your appointments; your treatment plan and medication; your physician's consultation notes; and your lab test results.

Would you use a patient portal to access your personal health information relative to your HIV care?

- ☐ Yes
- ☐ No
- ☐ I don't know

What is your level of comfort with accessing your personal health information on a smart device?

- ☐ I would like access to all my medical records as soon as the information is available.
- ☐ I would like access to all my medical records, after I have reviewed them with my doctor.
- ☐ I would like access to just my appointments and other need-to-know information (for example, instructions on how to go to the clinic).
- ☐ I do not want access to my medical records on a smart device.

The term health technology refers here to any health-related application (app) or software. Please indicate to which extent you agree with the following statements

	Strongly disagree	Somehow disagree	Disagree	Undecided	Agree	Somehow agree	Strongly agree
It is easy for me to use health technology	○	○	○	○	○	○	○
I have the capability to use health technology	○	○	○	○	○	○	○
I do not feel comfortable using health technology	○	○	○	○	○	○	○
When using health technology I worry I might press the wrong button and risk my health	○	○	○	○	○	○	○

Please leave additional comments here: ______________________

A patient-reported outcome measure is a questionnaire or survey about a given aspect of their health or illness, in which answers come directly from patients. They capture answers from patients, with no input from a physician or health professional. They are not blood test results (for example, viral load, CD4 counts).

Please specify your level of interest in filling out these different types of patient-reported outcome measures:

	Not at all interested	Not interested	Undecided	A little interested	Very interested	I don't know
Quality of Life	○	○	○	○	○	○
Experience of treatment (e.g., attitudes towards treatment, side effects, satisfaction with treatment)	○	○	○	○	○	○
Experience of healthcare (e.g., patient needs, quality of care, barriers to care)	○	○	○	○	○	○
Psychological challenges (e.g., stress, depression)	○	○	○	○	○	○
Symptoms (e.g., symptoms of HIV, fatigue, psychomotor slowness)	○	○	○	○	○	○
Psychological resources (e.g., perceived control, spiritual beliefs and activities, resiliency)	○	○	○	○	○	○
HIV self-management / self-care (e.g., adherence to treatment)	○	○	○	○	○	○
HIV-related stigma	○	○	○	○	○	○
Body and facial appearance (e.g., body image)	○	○	○	○	○	○
Social support (e.g., unsupportive social interactions)	○	○	○	○	○	○
Sexual and reproductive health (e.g., motivation for childbearing, HIV status disclosure, safer sex)	○	○	○	○	○	○
Disability	○	○	○	○	○	○
Others, please specify below:	○	○	○	○	○	○

If other, please specify

____

The staff administering this questionnaire will now show you different functions considered for the Opal application (app).

For each function considered for Opal, please specify how useful it is for you:

	Not at all useful	Not useful	Undecided	A little useful	Very useful	I don't know
Welcome Message, password	○	○	○	○	○	○
Calendar for appointments	○	○	○	○	○	○
Appointment Check-in	○	○	○	○	○	○
Map of appointment; indication of consultation room	○	○	○	○	○	○
Navigation; How to get to the hospital	○	○	○	○	○	○
Diagnosis	○	○	○	○	○	○
Notifications / Appointment reminder	○	○	○	○	○	○
Treatment Plan	○	○	○	○	○	○
Consultation Note	○	○	○	○	○	○
Possibility to share consultation notes	○	○	○	○	○	○
Education material	○	○	○	○	○	○
Education material - Booklets	○	○	○	○	○	○
Education material - Video	○	○	○	○	○	○
Lab results	○	○	○	○	○	○
Account Setting	○	○	○	○	○	○
Allow an alias/photo for the screen display	○	○	○	○	○	○

Please leave additional comments here:

____

Please indicate to which extent you agree with the following statements:

	Completely disagree	Somehow disagree	Somehow agree	Completely agree	I don't know
Opal meets my approval	○	○	○	○	○
Opal is appealing to me	○	○	○	○	○
I like Opal	○	○	○	○	○
I welcome Opal	○	○	○	○	○

Please indicate to which extent you agree with the following statements.

Using a secure electronic network, I am willing to allow my personal health information to be shared with...

	Completely disagree	Somehow disagree	Somehow agree	Completely agree	I don't know
...my primary HIV care provider	◯	◯	◯	◯	◯
... other clinicians at my HIV clinic.	◯	◯	◯	◯	◯
... the non-clinical staff at my HIV clinic.	◯	◯	◯	◯	◯
... non-HIV specialists	◯	◯	◯	◯	◯
... pharmacists.	◯	◯	◯	◯	◯
... my health insurers.	◯	◯	◯	◯	◯
... the local health department.	◯	◯	◯	◯	◯

Please indicate to which extent you agree with the following statements.

With Opal...

	Completely disagree	Somehow disagree	Somehow agree	Completely agree	I don't know
...I would better understand my health and medical conditions	◯	◯	◯	◯	◯
...I would better remember the plan for my care	◯	◯	◯	◯	◯
...I would take better care of myself	◯	◯	◯	◯	◯
...I would be more likely to take my medications as prescribed.	◯	◯	◯	◯	◯
...I would feel more in control of my health care.	◯	◯	◯	◯	◯
...I would be better prepared for visits.	◯	◯	◯	◯	◯
...I would worry more	◯	◯	◯	◯	◯
...I would be concerned about my privacy	◯	◯	◯	◯	◯
...the information would be more confusing than helpful	◯	◯	◯	◯	◯
...it could make my doctor's job more difficult	◯	◯	◯	◯	◯

Comments: ______________________________
