# Supplementary material for: Acceptability of a Patient Portal (Opal) in HIV Clinical Care: A Feasibility Study
Source: J Pers Med. 2021 Feb 16;11(2):134. doi: 10.3390/jpm11020134 (PMC7920437; doi:10.3390/jpm11020134)

Welcome password

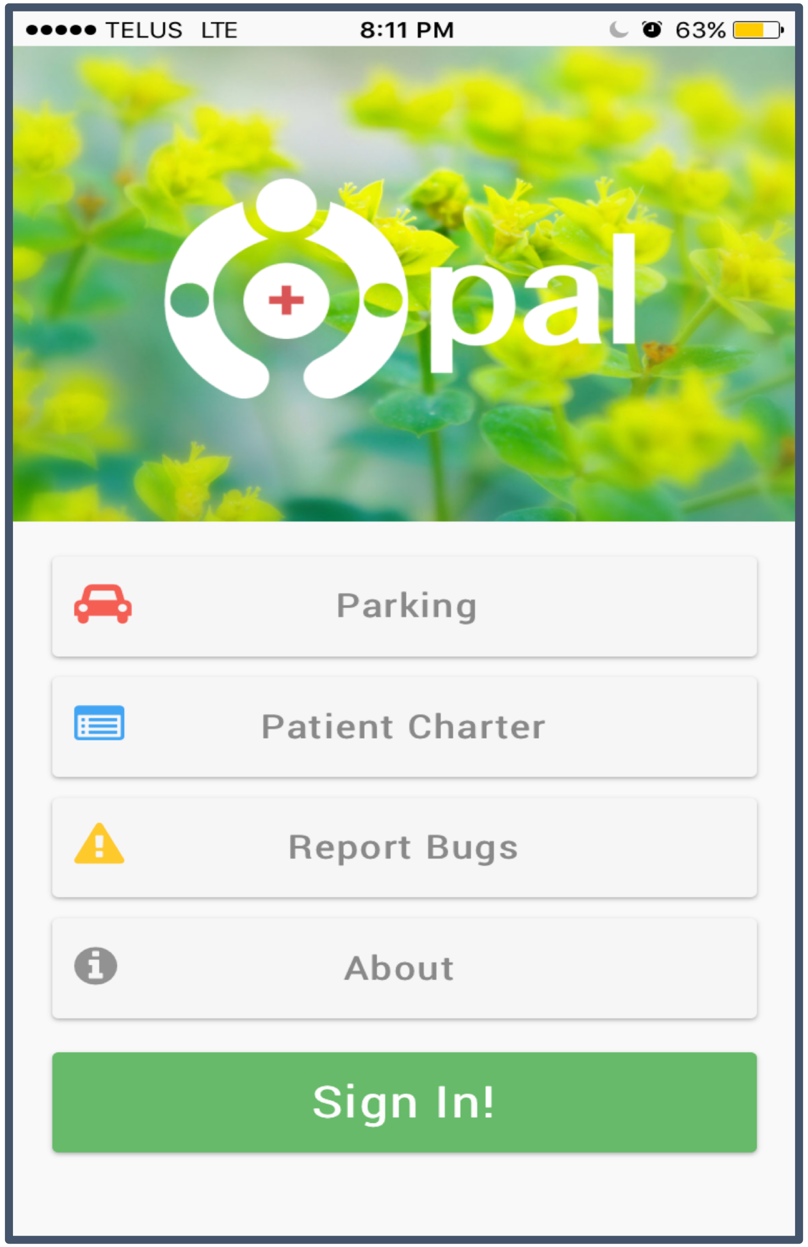

Calendar

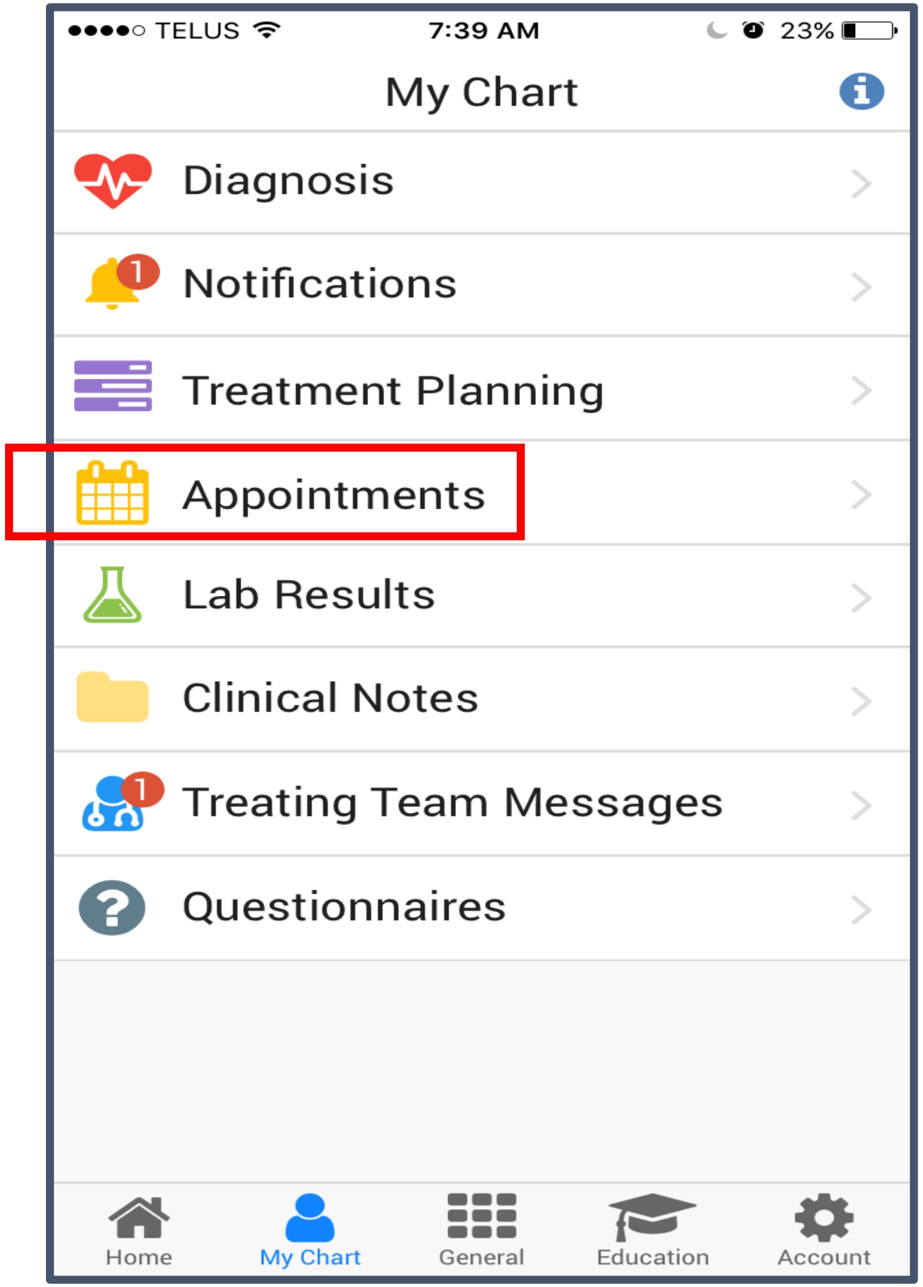

●●●●○ TELUS
7:39 AM
23%
My Chart
Diagnosis
Notifications
1
Treatment Planning
Appointments
Lab Results
Clinical Notes
Treating Team Messages
1
Questionnaires
Home
My Chart
General
Education
Account

Calendar

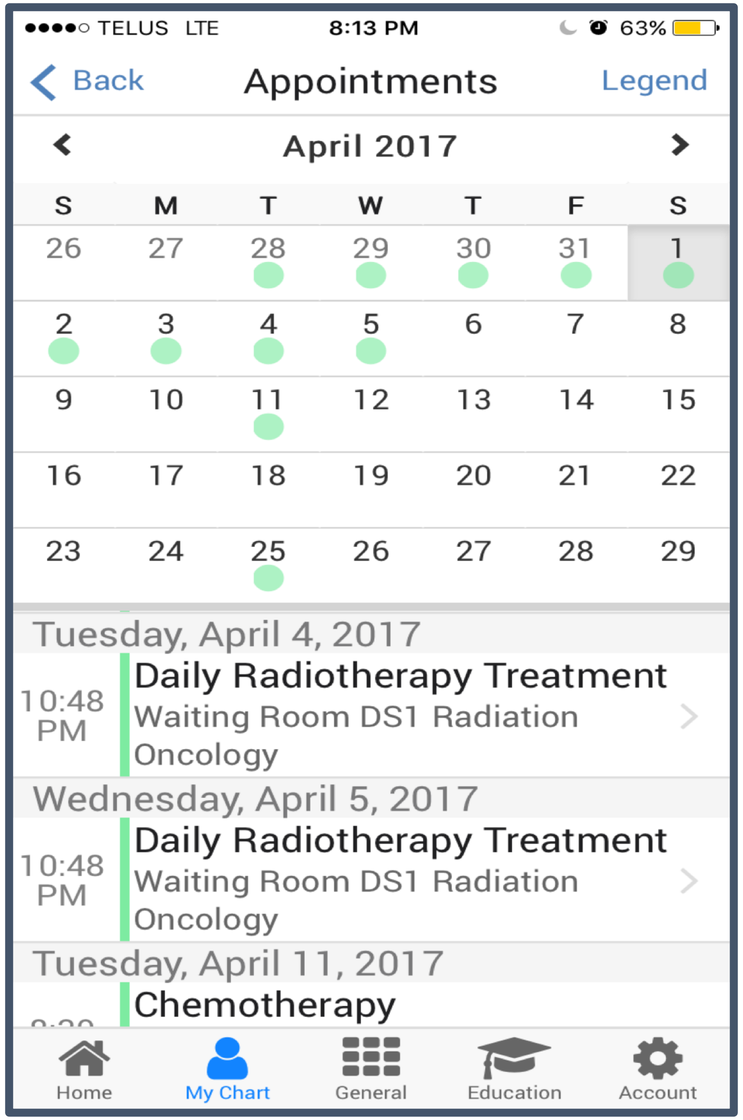

●●●●○ TELUS LTE
8:13 PM
63%
Back
Appointments
Legend
April 2017
S M T W T F S
26 27 28 29 30 31 1
2 3 4 5 6 7 8
9 10 11 12 13 14 15
16 17 18 19 20 21 22
23 24 25 26 27 28 29
Tuesday, April 4, 2017
10:48 PM
Daily Radiotherapy Treatment
Waiting Room DS1 Radiation Oncology
Wednesday, April 5, 2017
10:48 PM
Daily Radiotherapy Treatment
Waiting Room DS1 Radiation Oncology
Tuesday, April 11, 2017
Chemotherapy
Home
My Chart
General
Education
Account

Appointment check-in

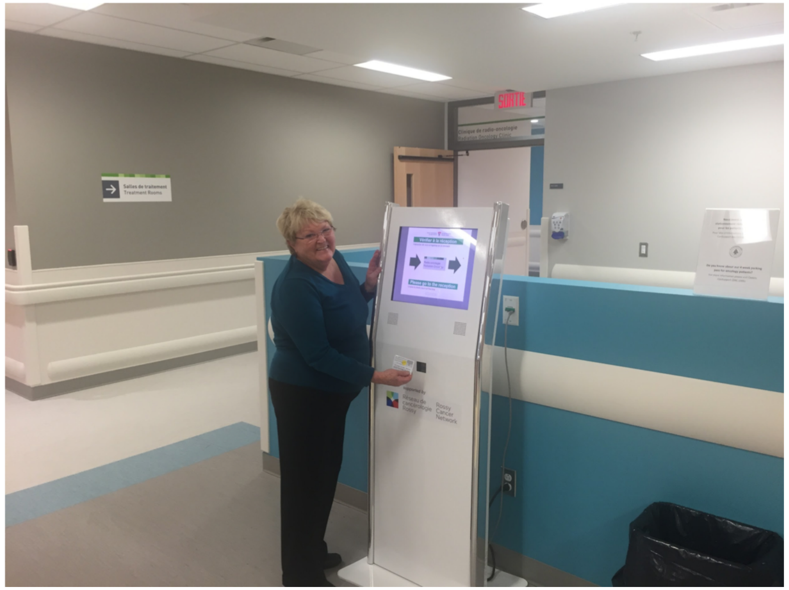

Virtual Waiting Room
Centre de traitement / Treatment Centre - Section A

Resource(s): Chemotherapy Treatment - Glen Treatment - Glen Bladder Installation
Location: TX AREA A

Patient Name	Scheduled	CheckIn	CheckIn Location	Wait (min)	Remaining (min)	Appointment	Resource	Primary Oncologist	Call Patient
BERNARD, LYSTRA (1164938)	14:45	14:34	RC Waiting Room	258	-248	CHM [Medivisit]	Chemotherapy Treatment - Glen	Chemotherapy Treatment - Glen	Call Patient
BURDAYRON, LINDA ROSE (0284680)	10:15	9:44	TEST CENTRE WAITING ROOM	548	-518	CHM [Medivisit]	Chemotherapy Treatment - Glen	Chemotherapy Treatment - Glen	Call Patient
GALEA, LYNDA (0977862)	10:00	9:43	TEST CENTRE WAITING ROOM	549	-533	TRANS [Medivisit]	Treatment - Glen	Treatment - Glen	Call Patient
SHECAPIO, CATHERINE (1251932)	0:00	9:05	RC Waiting Room	587	-1133	TRANS [Medivisit]	Treatment - Glen	Treatment - Glen	Call Patient

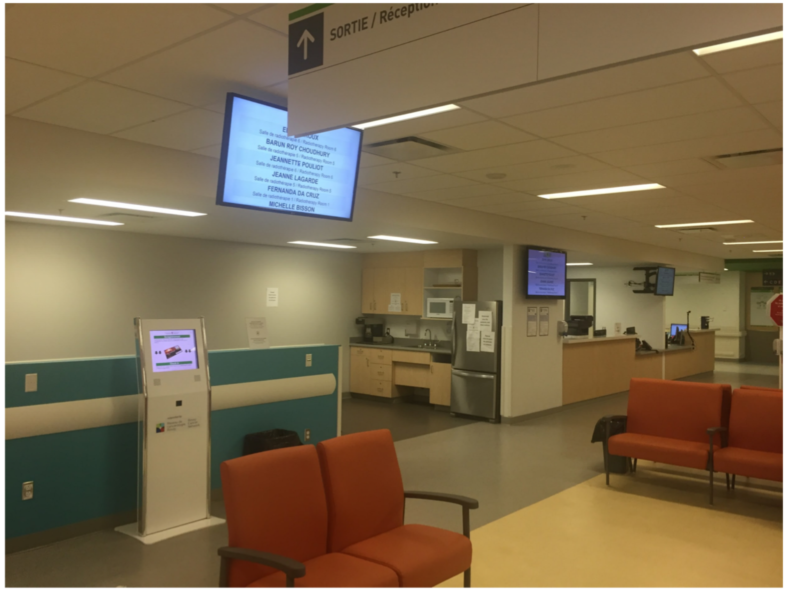

1) Check-in system
2) Virtual waiting room
3) Call-in system

1) Data -> Wait time predictions

Appointment check-in

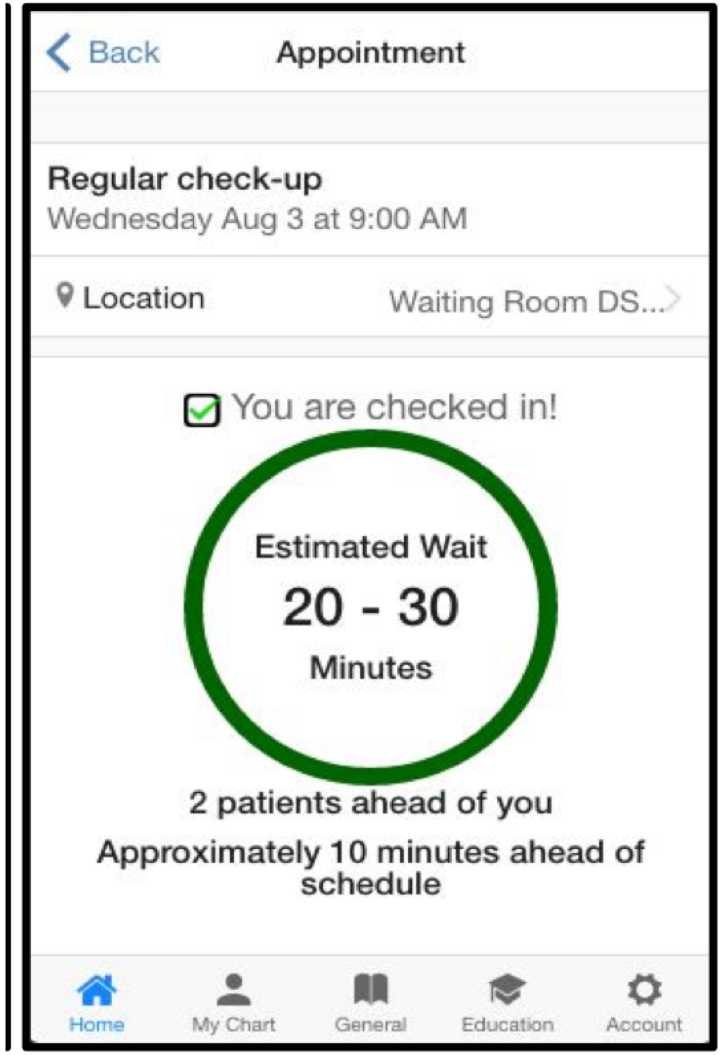

Navigation tool – Appointment location

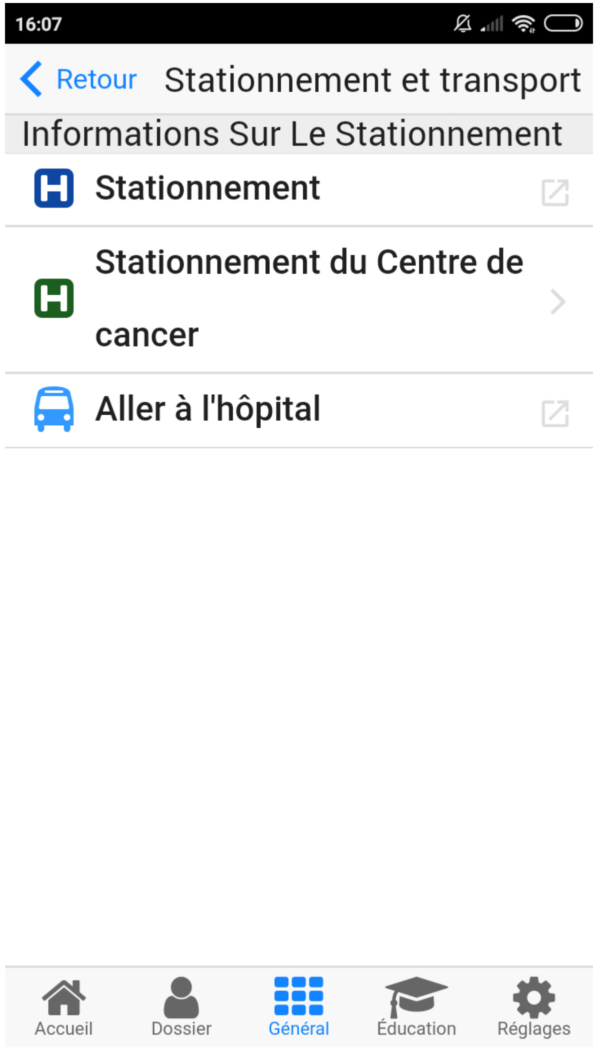

Navigation tool – Appointment location

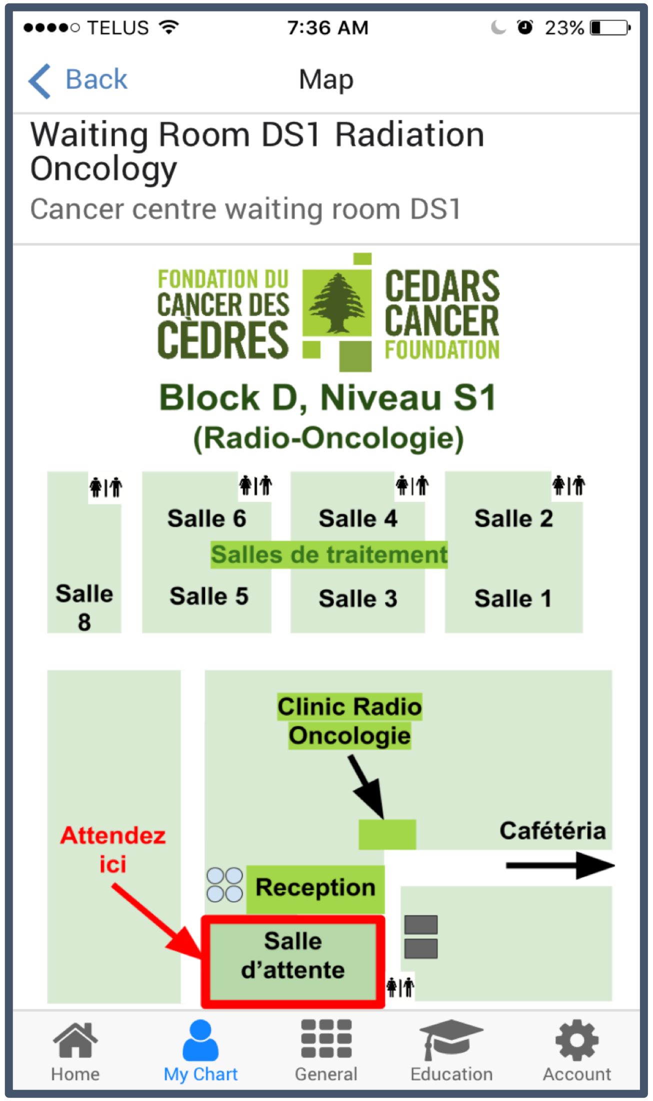

Diagnosis, lab results and treatment plan

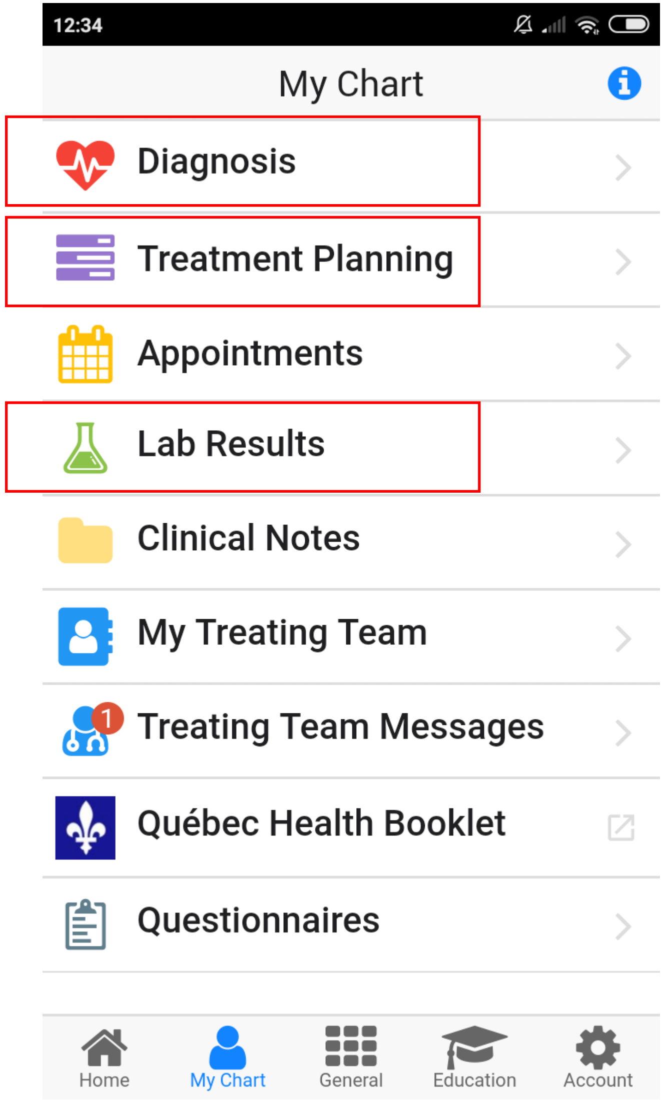

Diagnosis, lab results and treatment plan

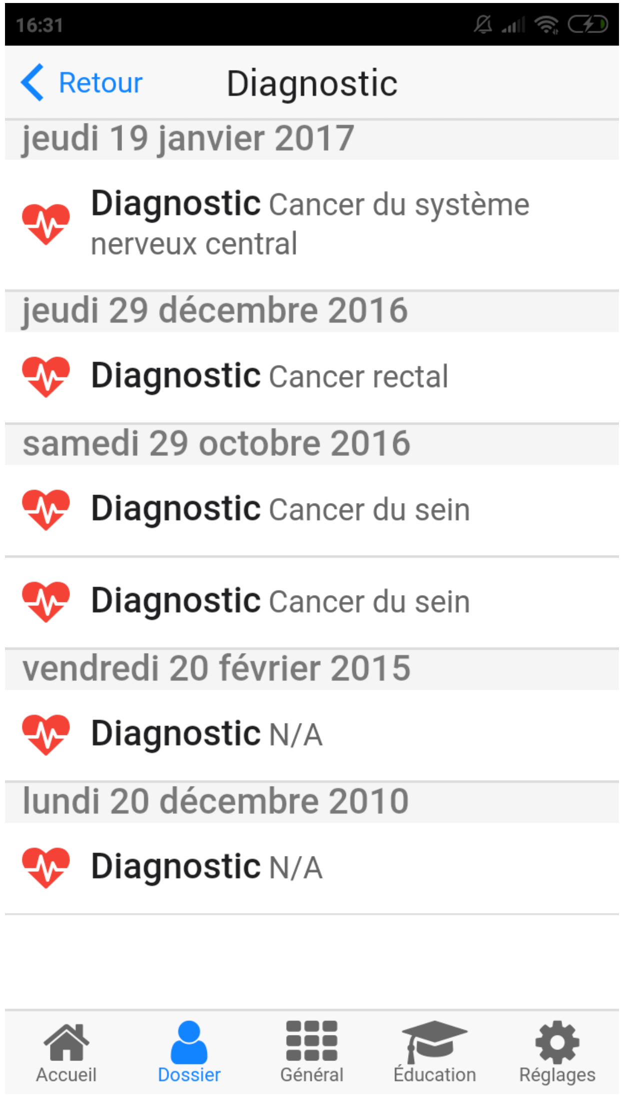

Diagnosis, **lab results** and treatment plan

by date…

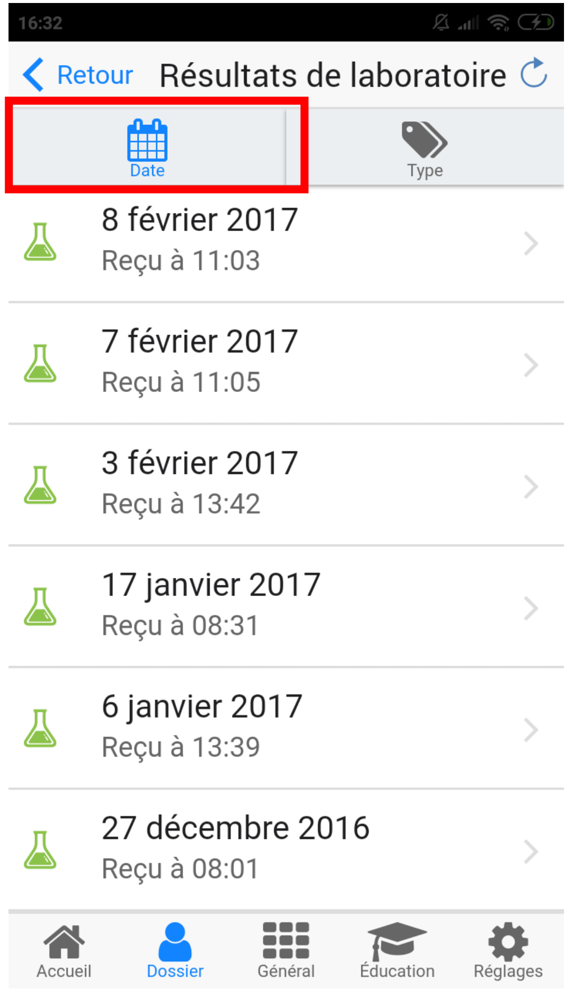

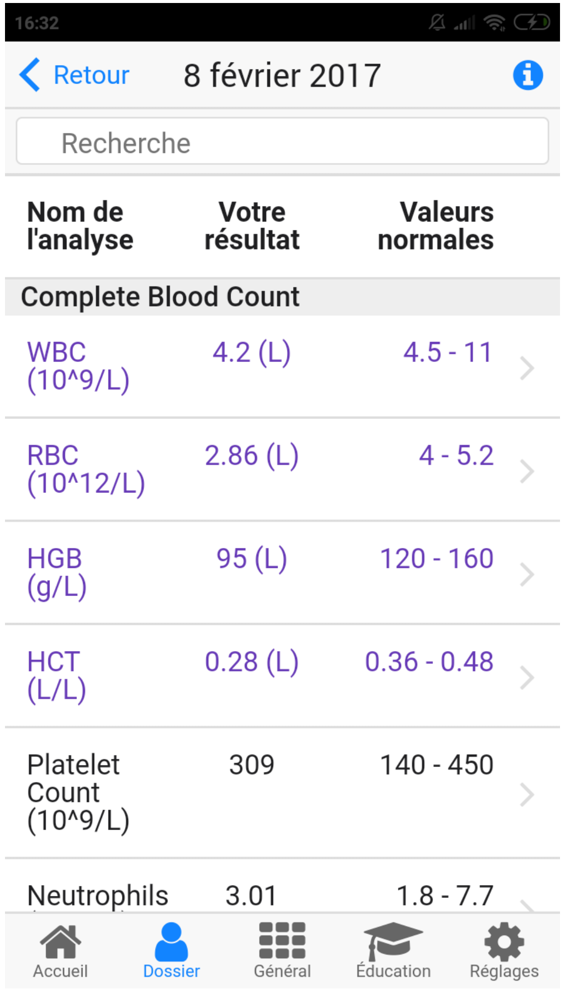

Diagnosis, **lab results** and treatment plan

by type…

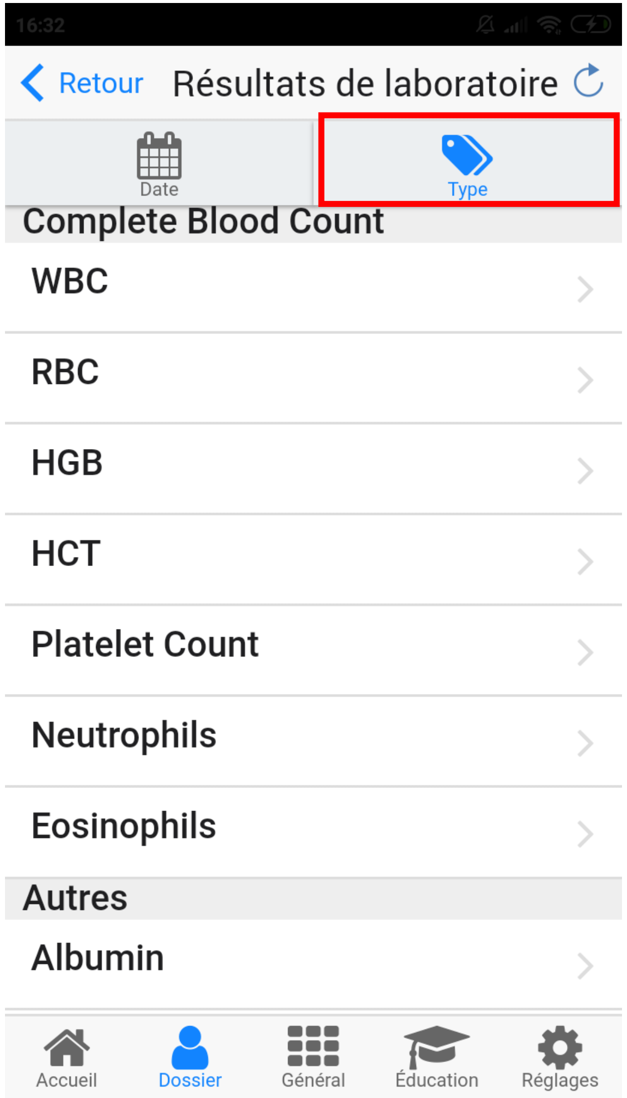

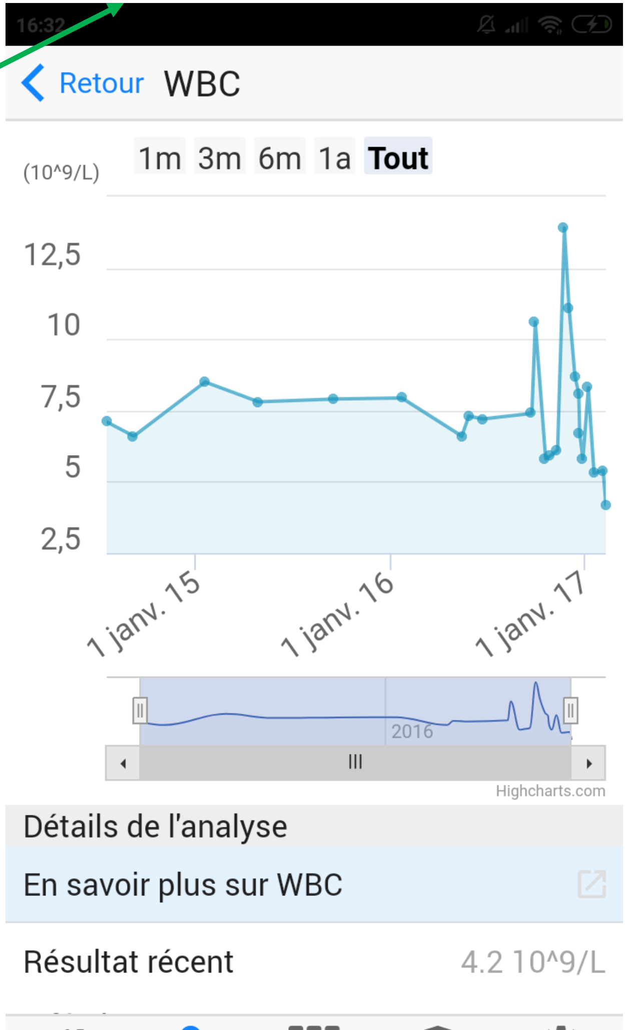

Diagnosis, lab results and **treatment plan**

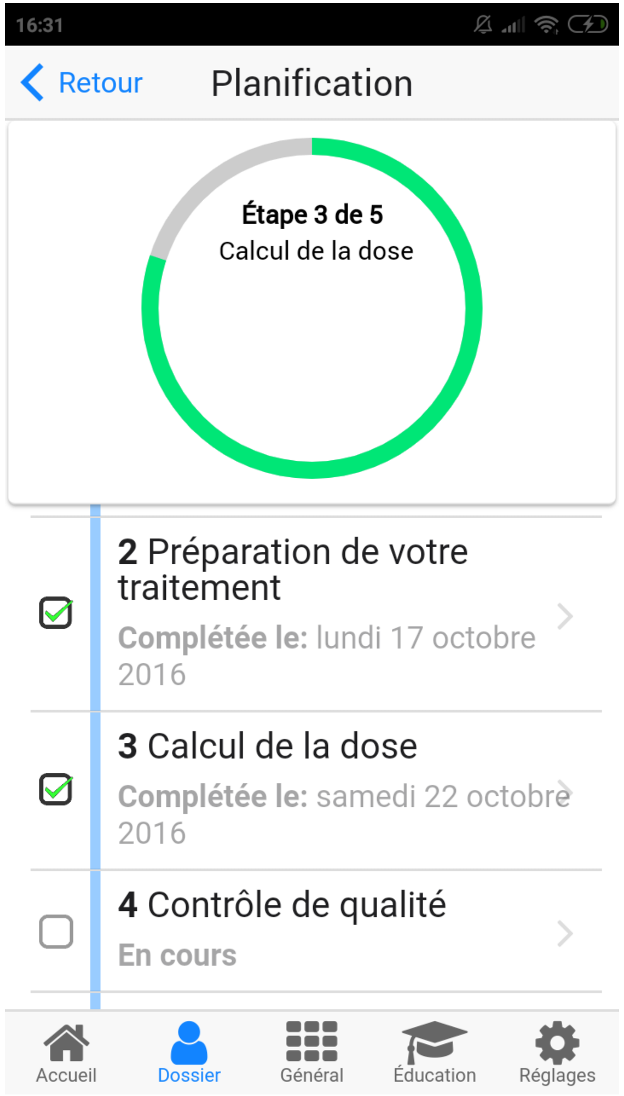

Diagnosis, lab results and treatment plan

*Is it **useful** for HIV care?*

*What **benefits** could this function have?*

*What **problems** could arise with this function?*

Consultation notes

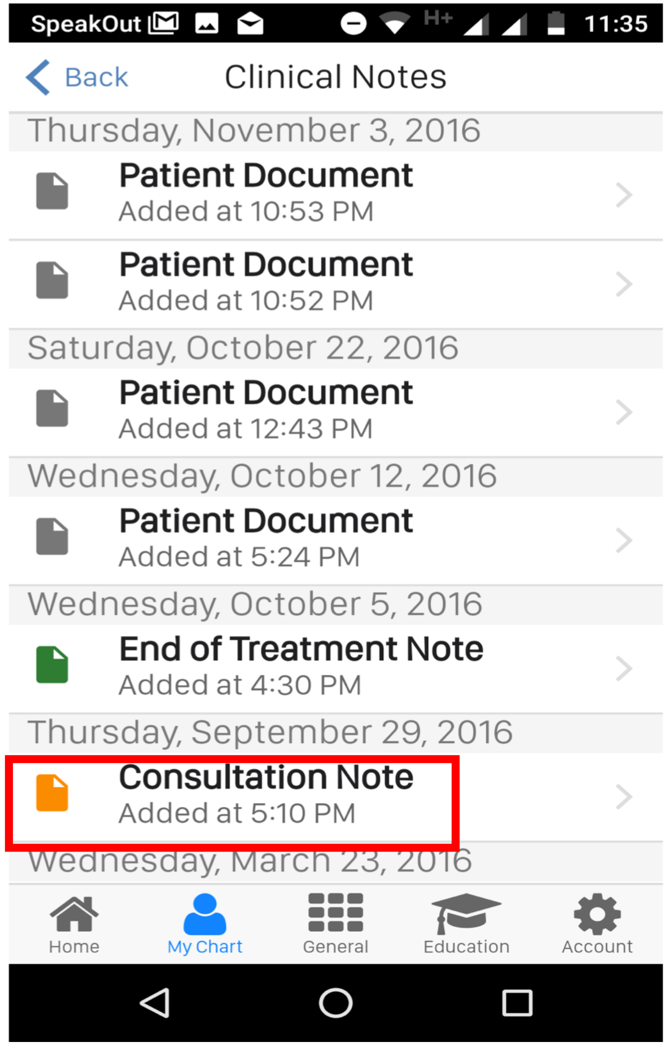

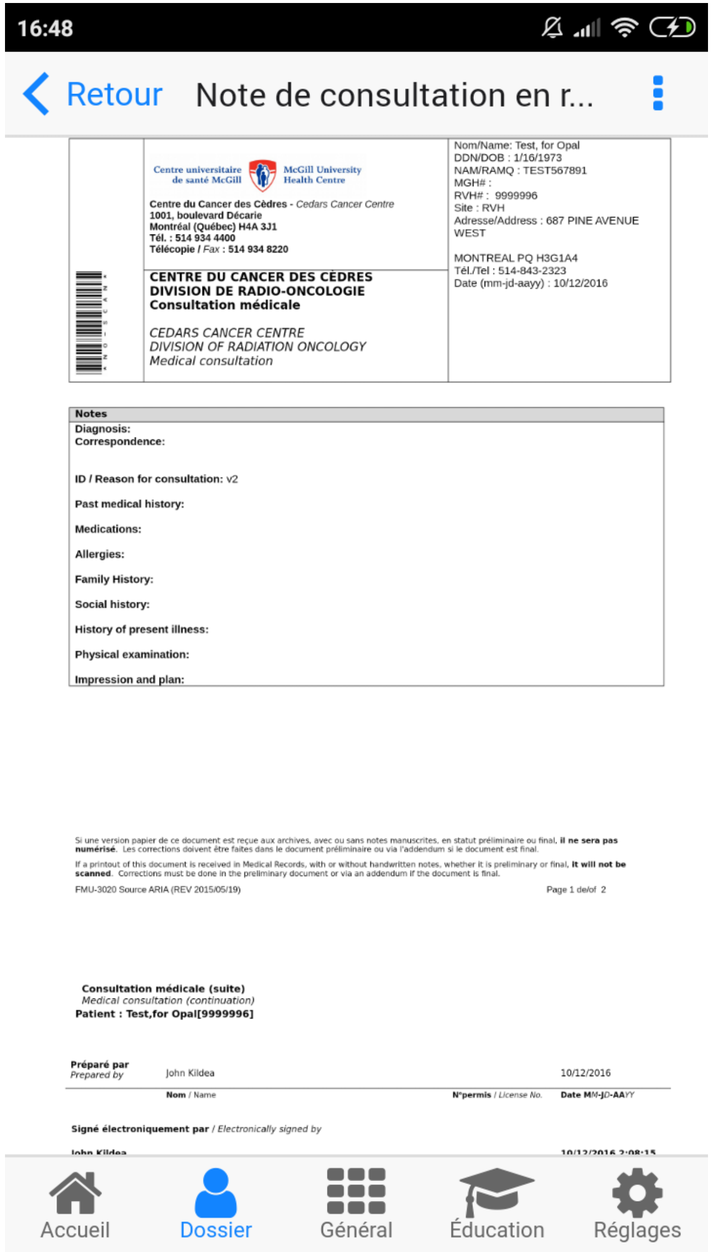

Consultation notes

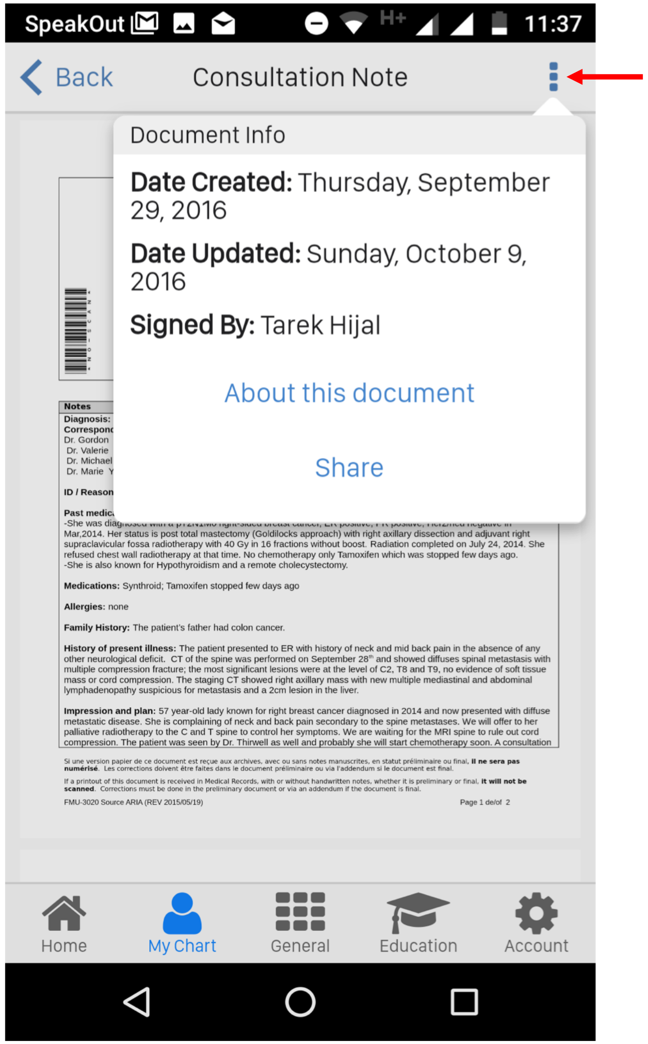

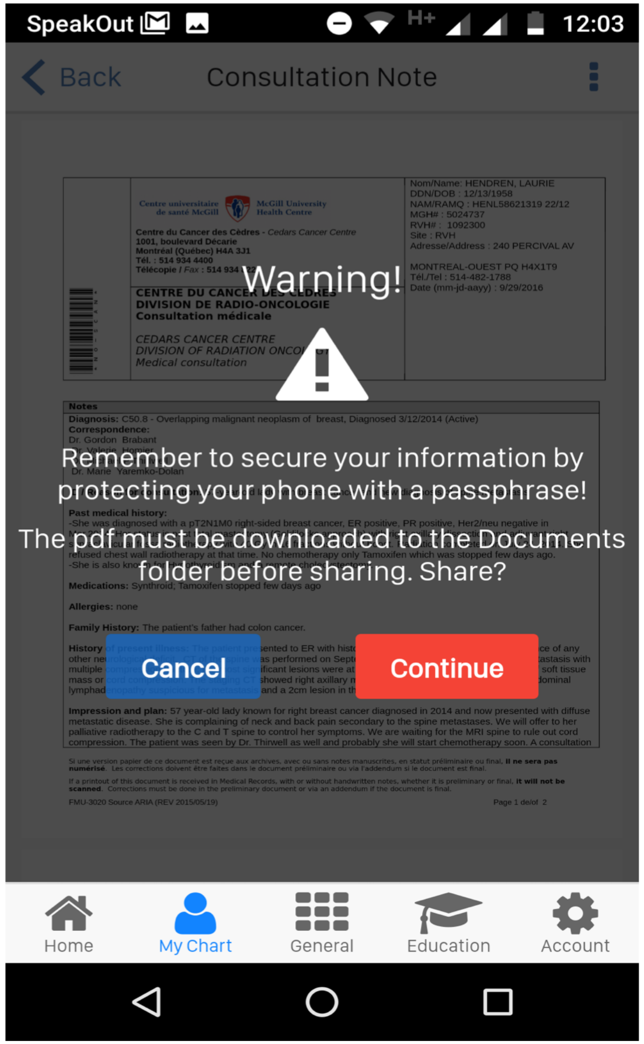

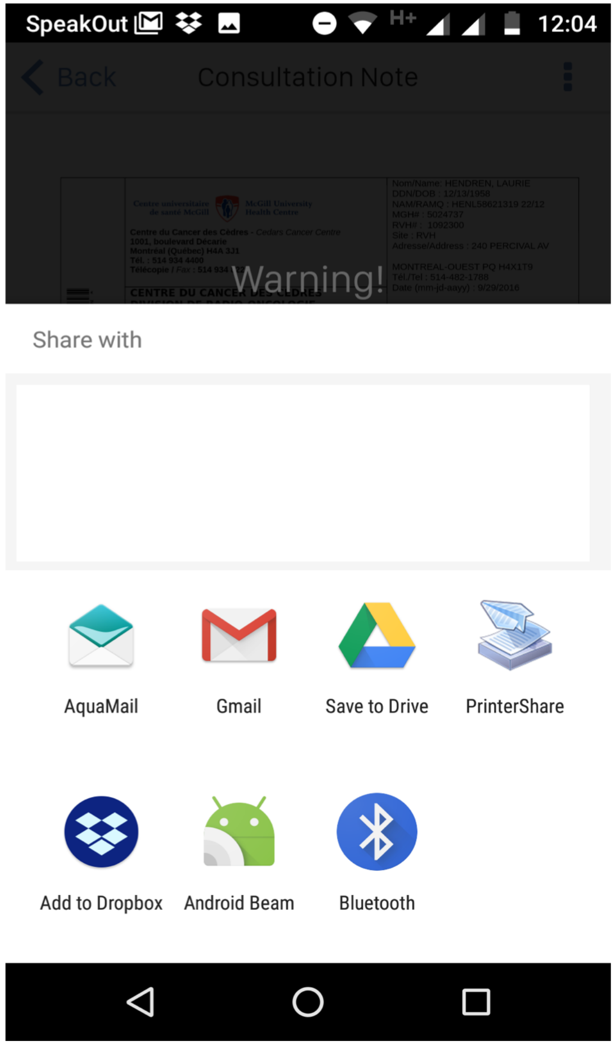

Educational material

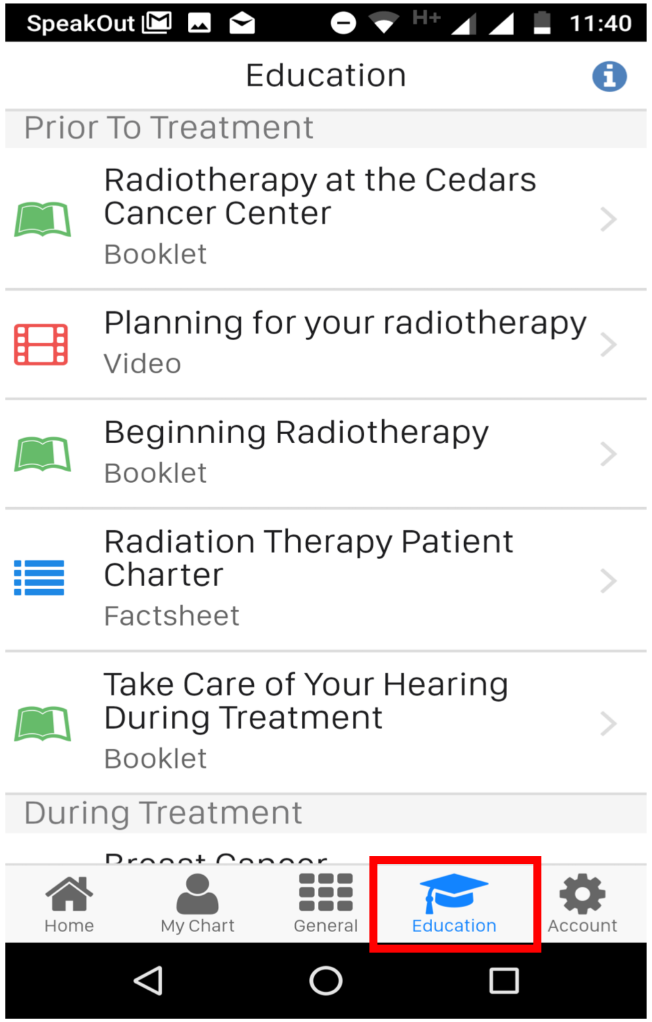

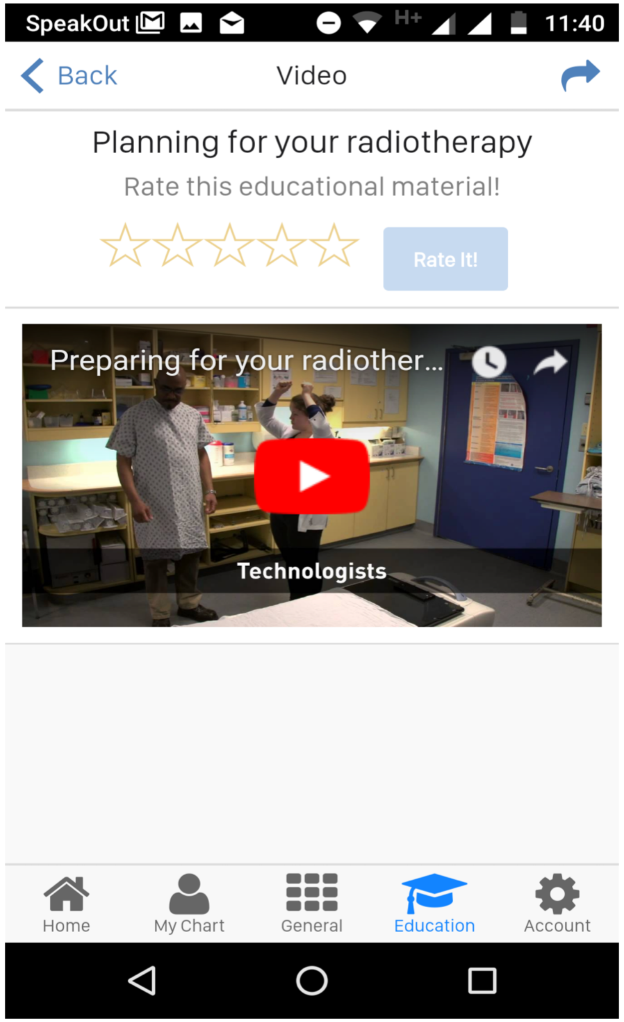

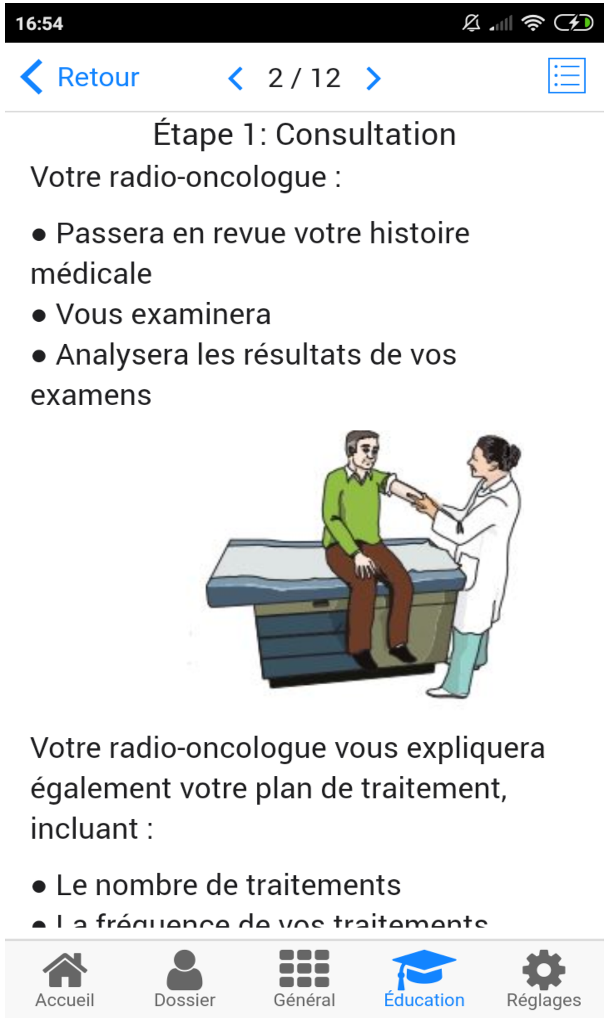

Account settings

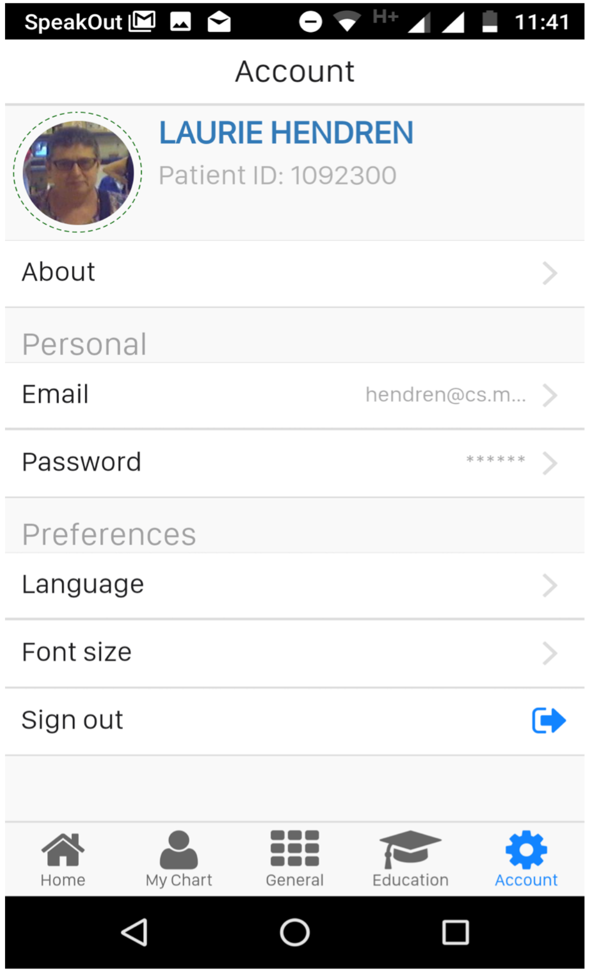

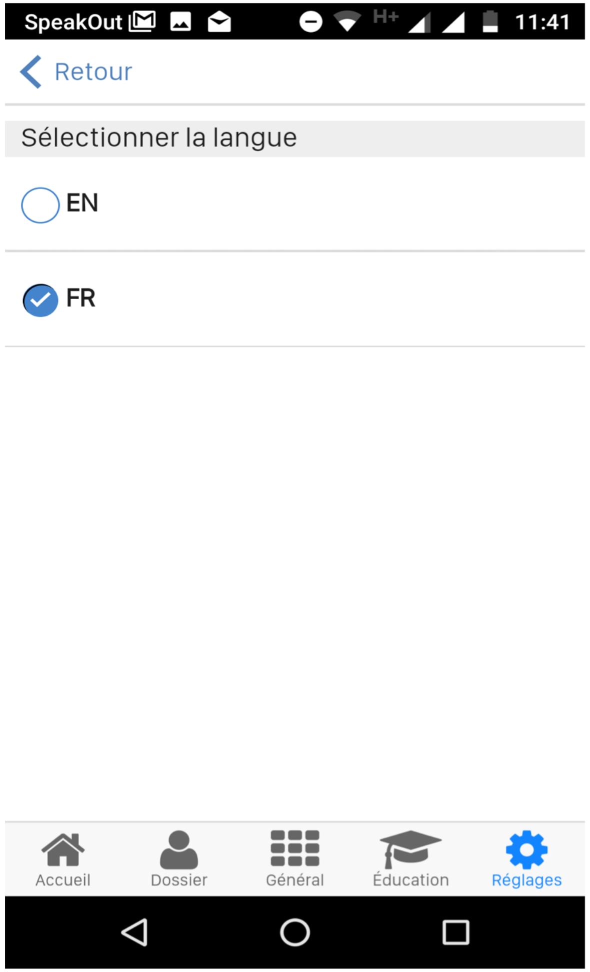

Supplement: Supplementary file 1 [file jpm-11-00134-s001.zip › S1_Presentation_Functions_Opal.pdf]
