# Supplementary material for: Acceptability of a Patient Portal (Opal) in HIV Clinical Care: A Feasibility Study
Source: J Pers Med. 2021 Feb 16;11(2):134. doi: 10.3390/jpm11020134 (PMC7920437; doi:10.3390/jpm11020134)
Supplement: Supplementary file 1 [file jpm-11-00134-s001.zip › S2_HCPs_Survey.pdf]

OPAL Adaptation for Healthcare Professionals

Record ID ______________________

Date ______________________

What is your date of birth? ______________________

What is your gender (select all that you apply)?
- ☐ Female
- ☐ Male
- ☐ Trans
- ☐ Other
- ☐ Prefer not to answer

If 'other', please specify ______________________

What is your occupation at the Chronic Viral Illness Service?
- ○ Physician
- ○ Nurse
- ○ Psychologist
- ○ Psychiatrist
- ○ Social Worker
- ○ Pharmacist
- ○ Other

If other, please specify ______________________

What is the first language that you learned? ______________________

What is your preferred language of communication when you provide HIV care?
- ☐ French
- ☐ English
- ☐ Other

If Other please specify. ______________________

Do you currently use any of the following smart devices? (check all that apply)
- ☐ Smartphone
- ☐ iPod, Phablet
- ☐ Tablet
- ☐ Smartwatch
- ☐ Computer, Laptop
- ☐ Other
- ☐ I do not use any of these devices

If other, please specify ______________________

Do you currently use an application (app) on a smart device for your work as an HIV care provider?
- ○ Yes
- ○ No

If yes, please specify: __________________________________

Do you know of any digital, smartphone, or web-based application (app) currently used by your HIV patients for their HIV health care?
- ◯ Yes
- ◯ No

If yes, please specify: __________________________________

How much experience do you have with health-related applications (apps) on smart devices?
- ◯ None
- ◯ Very Little
- ◯ Average
- ◯ Quite Extensive
- ◯ Very Extensive

A patient portal is a secure website or application (app) that gives you access to your medical records, as well as other services (for example, a mobile check-in function and messaging with care providers).

Patients' medical records include: their diagnosis; their personal and medical information; a list of their appointments; their treatment plan and medication; their physician's consultation notes; and their lab test results.

Would you use a patient portal for your HIV patients that allows them to access their medical records?
- ◯ Yes
- ◯ No
- ◯ I don't know

Please indicate to which extent you agree with the following statements.
Comments: __________________________________

	Strongly Disagree	Disagree	Somewhat Disagree	Undecided	Somewhat Agree	Agree	Strongly Agree
It is easy for me to use health technology.	◯	◯	◯	◯	◯	◯	◯
I have the capability to use health technology	◯	◯	◯	◯	◯	◯	◯
I do not feel comfortable using health technology	◯	◯	◯	◯	◯	◯	◯
When using health technology, I worry I might press the wrong button	◯	◯	◯	◯	◯	◯	◯

Please specify your level of interest in having your HIV patients fill out patient-reported outcome measures via Opal for each of the following types. __________________________________

	Not at all interested	Not Interested	Undecided	A little Interested	Very Interested	I dont know
Quality of Life	○	○	○	○	○	○
Experience of treatment (e.g., attitudes towards treatment, side effects, satisfaction with treatment)	○	○	○	○	○	○
Experience of healthcare (e.g., patient needs, quality of care, barriers to care)	○	○	○	○	○	○
Psychological challenges (e.g., stress, depression)	○	○	○	○	○	○
Symptoms (e.g., symptoms of HIV, fatigue, psychomotor slowness)	○	○	○	○	○	○
Psychological resources (e.g., perceived control, spiritual beliefs and activities, resiliency)	○	○	○	○	○	○
HIV self-management / self-care (e.g., adherence to treatment)	○	○	○	○	○	○
HIV-related stigma	○	○	○	○	○	○
Body and facial appearance (e.g., body image)	○	○	○	○	○	○
Social support (e.g., unsupportive social interactions)	○	○	○	○	○	○
Sexual and reproductive health (e.g., motivation for childbearing, HIV status disclosure, safer sex)	○	○	○	○	○	○
Disability	○	○	○	○	○	○

The staff administering this questionnaire will now show you different functions considered for the Opal application. ________________________________

For each function considered for Opal, please specify how useful it is for you:

	Not at all useful	Not useful	Undecided	A little useful	Very useful	I dont know
Welcome Message	○	○	○	○	○	○
Appointment Schedule	○	○	○	○	○	○
Appointment Check-in	○	○	○	○	○	○
Appointment Map	○	○	○	○	○	○
Navigation tool, top left	○	○	○	○	○	○
Contacts	○	○	○	○	○	○
Notifications	○	○	○	○	○	○
Treatment Plan	○	○	○	○	○	○
Consultation Notes	○	○	○	○	○	○
Messages	○	○	○	○	○	○
Education Material	○	○	○	○	○	○
Education Material - Booklets	○	○	○	○	○	○
Education Material - Videos	○	○	○	○	○	○
Notes	○	○	○	○	○	○
Account Setting	○	○	○	○	○	○
Allow an alias for the screen display	○	○	○	○	○	○

If you have any comments, please leave them here: ________________________________

Please indicate to which extent you agree with the following statements:

	Completely disagree	Somewhat disagree	Somewhat agree	Completely agree	I dont know
OPAL meets my approval	○	○	○	○	○
OPAL is appealing to me	○	○	○	○	○
I like OPAL	○	○	○	○	○
I welcome OPAL	○	○	○	○	○

If you have any comments, please leave them here: ________________________________

For physicians only:
Knowing that Opal may give patients access to their physician's consultation notes...

	I dont know	Completely disagree	Somewhat disagree	Somewhat agree	Completely agree
Patients will disagree with what I write in their visit notes	○	○	○	○	○
Patients will request changes to the content of visit notes	○	○	○	○	○
Patients will find significant errors in the notes	○	○	○	○	○

Patients will contact me or my practice with questions about their notes	◯	◯	◯	◯	◯

Please indicate to which extent you agree with the following statements:

	Strongly disagree	Somehow disagree	disagree	undecided	Agree	Somehow agree	Strongly agree
Using Opal is compatible with all aspects of my work	◯	◯	◯	◯	◯	◯	◯
Using Opal is completely compatible with my current situation	◯	◯	◯	◯	◯	◯	◯
I think that using Opal fits well with the way I like to work	◯	◯	◯	◯	◯	◯	◯
Using Opal fits into my work style	◯	◯	◯	◯	◯	◯	◯

Please leave any additional comments here. ______________________________
